# Proteomics-Driven Analysis of Ovine Whey Colostrum

**DOI:** 10.1371/journal.pone.0117433

**Published:** 2015-02-02

**Authors:** Domenica Scumaci, Francesca Trimboli, Ludovica Dell’Aquila, Antonio Concolino, Giusi Pappaianni, Laura Tammè, Giorgio Vignola, Alessia Luciani, Daniela Morelli, Giovanni Cuda, Andrea Boari, Domenico Britti

**Affiliations:** 1 Dpt. of Experimental and Clinical Medicine, Magna Græcia University of Catanzaro, Catanzaro, Italy; 2 Dpt. of Health Science, Magna Græcia University of Catanzaro, Catanzaro, Italy; 3 Faculty of Veterinary Medicine, University of Teramo, Teramo, Italy; 4 Istituto Zooprofilattico Sperimentale dell’Abruzzo e del Molise (IZSAM) “G. Caporale”, Teramo, Italy; Università della Calabria, ITALY

## Abstract

The aim of this study was to shed light in to the complexity of the ovine colostrum proteome, with a specific focus on the low abundance proteins. The ovine colostrum is characterized by a few dominating proteins, as the immunoglobulins, but it also contains less represented protein species, equally important for the correct development of neonates. Ovine colostrum, collected immediately after lambing, was separated by 1D SDS-PAGE. Proteins bands were digested with trypsin and the resulting peptides were analyzed by LC-MS/MS. On the basis of the Swiss-Prot database, a total of 343 unique proteins were identified. To our knowledge, this study represents the most comprehensive analysis of ovine colostrum proteome.

## INTRODUCTION

Colostrum is a complex biological fluid composed of water, proteins, carbohydrates, lipids, vitamins and minerals. Colostrum is secreted by the mammary gland immediately after parturition and provides nutrition, immunity and defense, and growth factors to the newborn [1]. The immunoglobulins are certainly the most important proteins of colostrum. In ruminants, the placentation types (epitheliochorial, cow; syndesmochorial, sheep) prevent the utero transfer of maternal immunoglobulins. For this reason, newborn ruminants rely on the ingestion and absorption of maternal immunoglobulins from colostrum [2–4]. This process, termed passive transfer, is important for subsequent protection against neonatal infectious diseases before development of their own adaptive immunity and other post-partum environmental challenges [4, 5].

Lambs born with a negligible serum IgG concentration, so neonatal lambs depend on the passive transfer of maternal IgG in colostrum to provide humoral immunity during neonatal period. Failure of the neonatal lambs to obtain and absorb colostral IgG has been linked to increase risk of illness, death from bacterial septicemia, common neonatal diseases and impaired growth performance [2, 4; 6–9]. On this basis, colostrum proteins can be divided into two major categories: i) high abundance proteins, mainly immunoglobulins and caseins, and, ii) a wide range of low abundant proteins. In this category are included proteins that contribute to protection of newborns against bacterial and viral infections [10, 11] and other postpartum environmental challenges such as complement factors, acute-phase proteins, anti-microbial proteins and peptides, and cytokines [12, 13], and proteins that contribute to the nutrition and to the development of various parts of newborn organism, such as growth-promoting components, important for development of gastrointestinal tract [14, 15].

The biological properties of other low abundance proteins are yet to be determined, but it is interesting to report that bovine colostrum proteins have beneficial effects on some human pathologies, as tumor or neurodegenerative diseases, like Alzheimer’s [16]. Many authors have demonstrated that dietary whey proteins could prevent tumors by increasing glutathione levels in serum and tissues as well as enhancing splenic lymphocyte proliferation, T helper and cytotoxic T cell activity [16]. Moreover, some researchers suggest that also β-lactoglobulin, α-lactalbumin, serum albumin, and lactoferrin could have anticancer potential [16]. Lactoferrin, in particular, inhibits intestinal tumors and perhaps tumors in other organs stimulating apoptosis, inhibiting angiogenesis and modulating carcinogen metabolizing enzymes [16]. The principal obstacle to the detailed study of low abundance proteins in colostrum is the high number of these proteins. In the last decade, proteomics has been established as a reliable and successful approach for the study of complex biological fluids, representing a powerful tool for the simultaneous analysis of hundred proteins in complex mixtures.

Several proteomics studies have been performed on mammalian colostrum and milk, e.g. human [17, 18], sow [19], mare [20], and especially bovine [21–24]. In these studies, proteomics has been applied to differentiate between healthy and mastitic bovine milk response of the mammary gland to various pathogens [25]. Senda et al. investigated changes in bovine whey proteome during the first ten days after calving [24], demonstrating that most of the low abundance proteins in colostrum relate to the passive immunity of neonates and some of them are important to their nutrition [22].

In an elegant study, Nissen et al. performed an extensive fractionation of colostrum prior to 2D-LC-MS/MS analysis, to gain a comprehensive picture of the bovine colostrum; this original approach brought to the identification of 403 proteins, which is, by far, the most extensive list of bovine colostrum proteins available in the literature [26].

In another study by Chiaradia et al. on ovine milk, healthy and subclinical mastitic ovine milk and MFG were analyzed to unveil a proteomic pattern that could be used as a putative sub-mastitis biomarker [27]. Currently little is known about low abundance proteins in ovine colostrum and their biological properties.

The aim of this work is to generate a map of the low abundance proteins expressed in ovine whey colostrum that will allow a better understanding of how colostrum may influence the health and the growth of lambs.

## MATERIALS AND METHODS

### Animals and colostrum collection

All colostrum samples were obtained from 15 Appenninic sheep [3–4 years old; 6–8 lactations) that were provided by a commercial farm placed in a mountainous area of the Abruzzo Region, Italy.

The project has been approved by the Committee on Animal Research and Ethics of the Universities of Chieti-Pescara, Teramo and Experimental Zooprophylactic Institute of AeM (CEISA) (http://www.unich.itlunichieti/appmanagerlfederati/CEISA).

The sheep were selected on the basis of clinical examination and following restriction: i) regular vaccinations and deworming; ii) absence of infectious diseases, postpartum infections, and compromised pregnancy; iii) normal births/deaths ratio; iv) normal performance of weaning. No animals had pharmacological treatments during the previous month before samples collection.

During summer of 2012 and winter of 2013, fifteen colostrum samples, approximately 30 mL each, were collected immediately after parturition from an half of the udder by hand, brought immediately to the laboratory, and immediately stored at -80°C until use, according to proteomics good practice guidelines.

### Whey preparation

To separate the cream and the skimmed fractions, colostrum samples were centrifuged at 3000 x g at 4°C for 30 min (Megafuge 1.0R; Hereus). To precipitate the casein fraction, skimmed colostrum was centrifuged at 100000 × g at 4° C for 60 min (Optima L-90K Ultracentrifuga; Beckman Coulter). Bright supernatant was filtered with a syringe filter 0,45 μm (Minisart, Sartorius stedim), mixed with protease inhibitor cocktail (Sigma Aldrich) and stored at −20° C until use.

### Determination of protein concentration and pooling of samples

Protein determination was performed with the purpose of pooling whey samples and for 2D-LC-MS/MS sample preparation. Protein concentrations from each whey sample were quantified in triplicate by the Bradford assay (Bio-Rad protein assay, Bio-Rad Laboratories GmbH) using BSA (Bovine Serum Albumin, Sigma Aldrich) as standard, according to the manufacturer’s instructions.

For the analysis, 15 whey samples were pooled on a proteins concentration (μg/μl) basis, in the attempt to create a single pool with a minimal intra- sample variability.

### 1D SDS-PAGE analysis and protein digestion

50 μg of 15 whey colostrum pooled samples were resuspended in Laemmli buffer [28], boiled, loaded into “Any kD” precast polyacrylamide gels (Bio-Rad Laboratories, Hercules, CA), subjected to electrophoresis (80 V, 2 hours), and stained with Coomassie blue [29].

**Fig 1:**
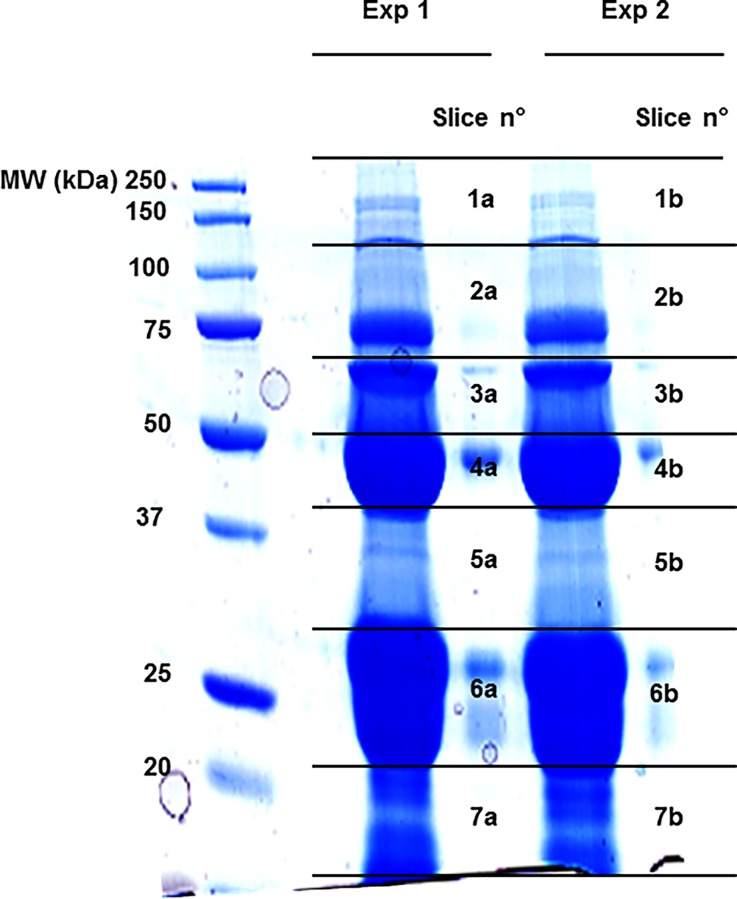
1d-SDS-PAGE. Proteins were resolved by Any kD Mini-PROTEAN TGX precast polyacrylamide gels. Gel line was sliced up in 7 pieces; gel bands were destained, reduced, alkylated, and digested with trypsin; peptides were resuspended and analyzed by LC-MS/MS.

Each gel lane was cut into 7 slices (Fig. 1), gel pieces were punched out manually, placed in a silicon Eppendorf tube and subjected to in-gel tryptic digestion as previously described [30].

Briefly, the gel slices were destained, washed, reduced, alkylated, and digested with trypsin; peptides were subsequently extracted, dried, and resuspended with 40 μL of 70% acetonitrile. The resulting tryptic peptides were purified by Pierce C18 Spin Columns (Thermo Fisher Scientific Inc.) according to the manufacturer’s procedure, eluted with 40 μL of 70% acetonitrile and dehydrated in a vacuum evaporator. Each purified tryptic peptide was analyzed through Nanoscale LC-MS/MS.

### Nanoscale liquid chromatography tandem mass spectrometry (LC-MS/MS) analysis

LC-MS/MS analysis was performed using an Easy LC 1000 nanoscale liquid chromatography (nanoLC) system (Thermo Fisher Scientific, Odense, Denmark) as described previously [31]. Briefly, peptide mixtures were loaded at 500 nL/min directly onto the analytical column; the analytical nanoLC column was a pulled fused silica capillary, 75 μmi.d., in-house packed to a length of 10 cm with 3 μm C18 silica particles from Dr. Maisch (Entringen, Germany). A binary gradient was used for peptide elution. Mobile phase A was 0.1% formic acid, 2% acetonitrile, whereas mobile phase B was 0.1% formic acid, 80% acetonitrile. Gradient elution was achieved at 350 nL/min flow rate, and ramped from 0% B to 30% B in 15 minutes, and from 30% B to 100% B in additional 5 minutes; after 5 minutes at 100% B, the column was re-equilibrated at 0% B for 10 minutes before the following injection. MS detection was performed on a quadrupole-orbitrap mass spectrometer Q-Exactive (Thermo Fisher Scientific, Bremen, Germany) operating in positive ion mode, with nanoelectrospray (nESI) potential at 1800 V applied on the column front-end via a tee piece. Data-dependent acquisition was performed by using a top-12 method with resolution (FWHM), AGC target and maximum injection time (ms) for full MS and MS/MS of, respectively, 70,000/17,500, 1e6/5e5, 50/400. Mass window for precursor ion isolation was 2.0 m/z, whereas normalized collision energy was 30. Ion threshold for triggering MS/MS events was 2e4. Dynamic exclusion was 15 s. Data were processed with Proteome Discoverer 1.3 (Thermo Fisher Scientific, Bremen, Germany), using Sequest as search engine, and the Ovis aries (Sheep)-uniprot-organism fasta as sequence database. Data were analyzed using the Mascot MudPIT search in Mascot Daemon. The following search parameters were used: MS tolerance 15 ppm; MS/MS tolerance 0.02 Da; fixed modifications: carbamidomethylation of cysteine; variable modification: oxidation of methionine, phosphorylation of serine, threonine and tyrosine; enzyme trypsin; max. missed cleavages 2; taxonomy Human. Protein hits based on two successful peptide identifications (Xcorr> 2.0 for doubly charged peptides, >2.5 for triply charged peptides, and >3.0 for peptides having a charge state >3) were considered valid. The false discovery rate (FDR) against reversed decoy database was below 2%.

### Protein Categorization

The identified whey colostrum proteins were classified based on the PANTHER (Protein ANalysis THrough Evolutionary Relationships) system (http://www.pantherdb.org), a unique resource that classifies genes and proteins by their functions. Comparisons among classes were plotted using Microsoft Excel (Microsoft Office 2010) [32].

### IPA analysis

Ingenuity Pathway Analysis, version 7 (IPA; Ingenuity Systems, USA; www.analysis.ingenuity.com) was performed to identify the molecular pathways and functional groupings based on published literature for the significant proteins.

Sheep UniProt IDs were replaced with the UniProt IDs for the closest human protein equivalents in order to enable the exploitation of the knowledge-based IPA software [33]

The list of protein identifications (IDs) was imported into the online software package IPA (Version 18030641, 12/07/2013) to determine their biological processes, functions, pathways, and molecular networks; analyses were performed with thresholds of 0.05 for P value; both direct and indirect relationships were considered.

## RESULTS AND DISCUSSION

The aqueous whey fraction of ovine colostrum was isolated by removing the fat layer, and casein precipitate by ultracentrifugation.

1D SDS-PAGE was used for separation of the proteins prior to LC- MS/MS analysis (Fig. 1). Two distinct experiments, in which peptides extracted from a PAGE slice were analyzed separately, were performed. Mass spectrometry data were processed in MudPIT mode. As expected, in both replicates there was a high consistency in the number of peptides and proteins.

In this study, a total of 342 proteins were identified (S1 Supporting Information). The fractionation method we applied resulted to be extremely efficient for protein separation, allowing the identification of 2652 non-redundant peptides.

The identified proteins were classified in accordance with their molecular function and the biological process by using the PANTHER classification system.

**Fig 2:**
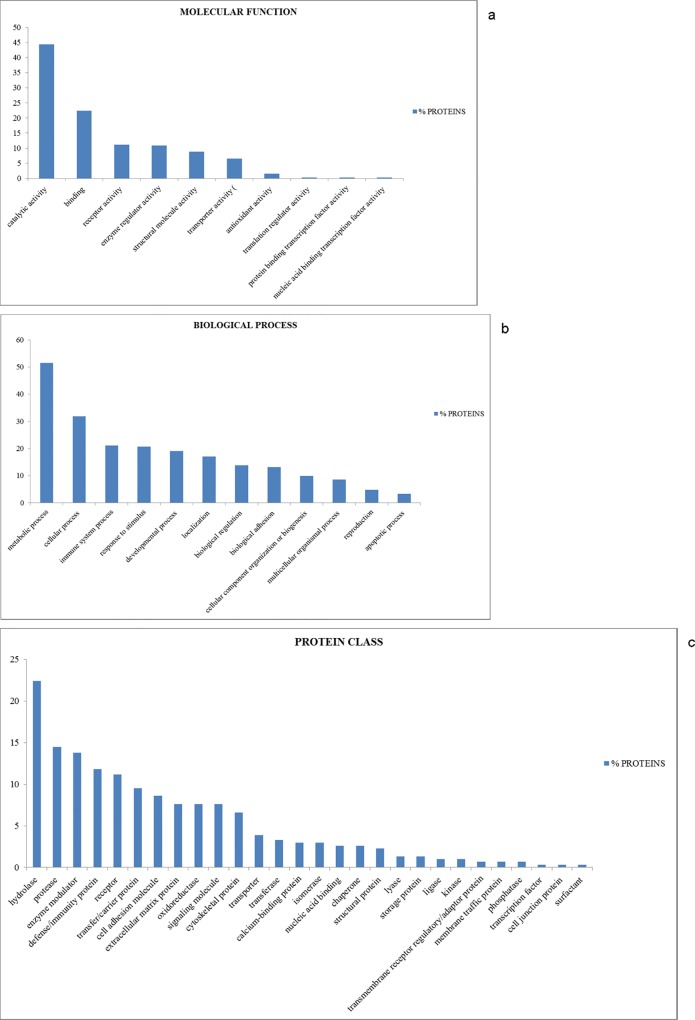
Gene Ontology annotation of protein. To have a general picture of the molecular functions (a), biological processes (b), and protein class (c), of identified proteins, the Protein ANalysis THrough Evolutionary Relationships (PANTHER) Database was used.

As shown in Fig. 2, the whey colostrum proteins can be classified into 10 groups with different molecular functions (Fig. 2A), thus providing a comprehensive overview for the whey colostrum proteome in sheep.

About 44% of the whey colostrum proteins resulted to be involved in catalytic activity, representing the largest proportion of all the identified proteins. The second largest category refers to binding activity (22.4%), followed by receptor activity (11.2%), enzyme regulator activity (10.9%), structural molecule activity (8.9%), transporter activity (6.6%), antioxidant activity (1.6%), translation regulator activity (0.3%), protein binding transcription factor activity (0.3%) and nucleic acid binding transcription factor activity (0.3%).

Whey colostrum proteins were further categorized into 12 biological process groups (Fig. 2B). The largest group was related to metabolic process (51.6%), followed by cellular process (31.9%) immune system process (21.1%) response to stimulus (20.7%) and developmental process (19.1%).

Further analysis performed with the PANTHER software revealed that whey colostrum proteins could be classified into 28 distinct groups (Fig. 2C), the top ten of which are i) hydrolase (22.4%), ii) protease (14.5%), iii) enzyme modulator (13.8%), iv) defense/immunity protein (11.8%), v) receptor (11.2%), vi) transfer/carrier protein (9.5%), vii) cell adhesion molecule (8.6%), viii) extracellular matrix protein (7.6%), ix) oxidoreductase (7.6%), x) signaling molecule (7.6%).

Data obtained from LC-MS/MS analysis of sheep whey colostrum were uploaded into IPA software and overlaid onto a global molecular network developed from information contained in the application. Networks of these proteins were generated by IPA based on their connectivity, each ranked by a score. This score is based on the hypergeometric distribution, calculated with the right-tailed Fisher’s Exact Test, and corresponds to the negative log of this p-value. Functional analysis in IPA identified the published biological functions that were most significantly associated with the genes in the network. Genes or gene products are represented as nodes, where shape indicates functional groups, and the biological relationship between two nodes is represented as an edge (line). All lines are supported by at least one reference in literature, textbook, or from canonical information stored in the Ingenuity Pathways knowledge database.

**Fig 3:**
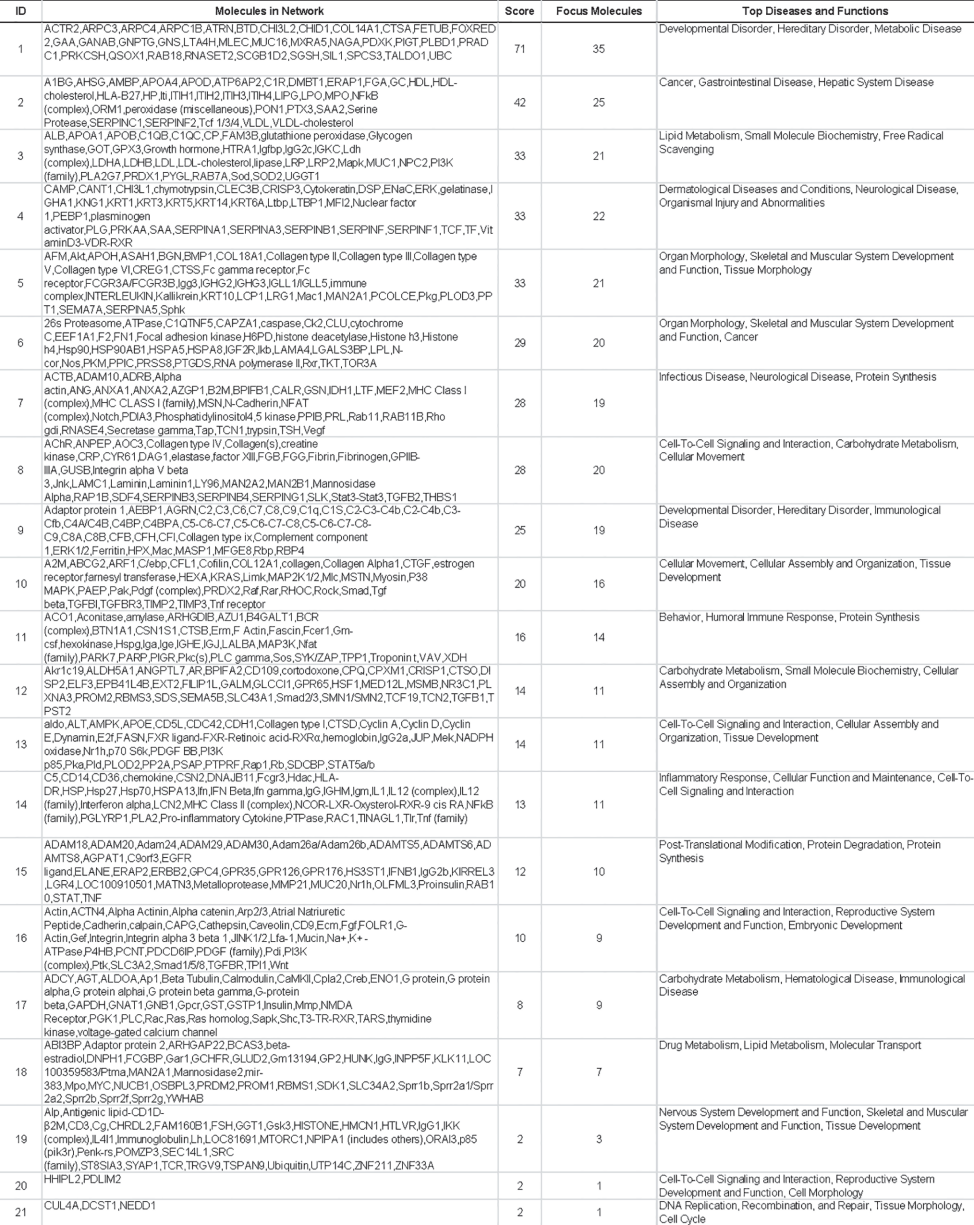
IPA analysis. List of signaling networks associated with identified proteins.

**Fig 4:**
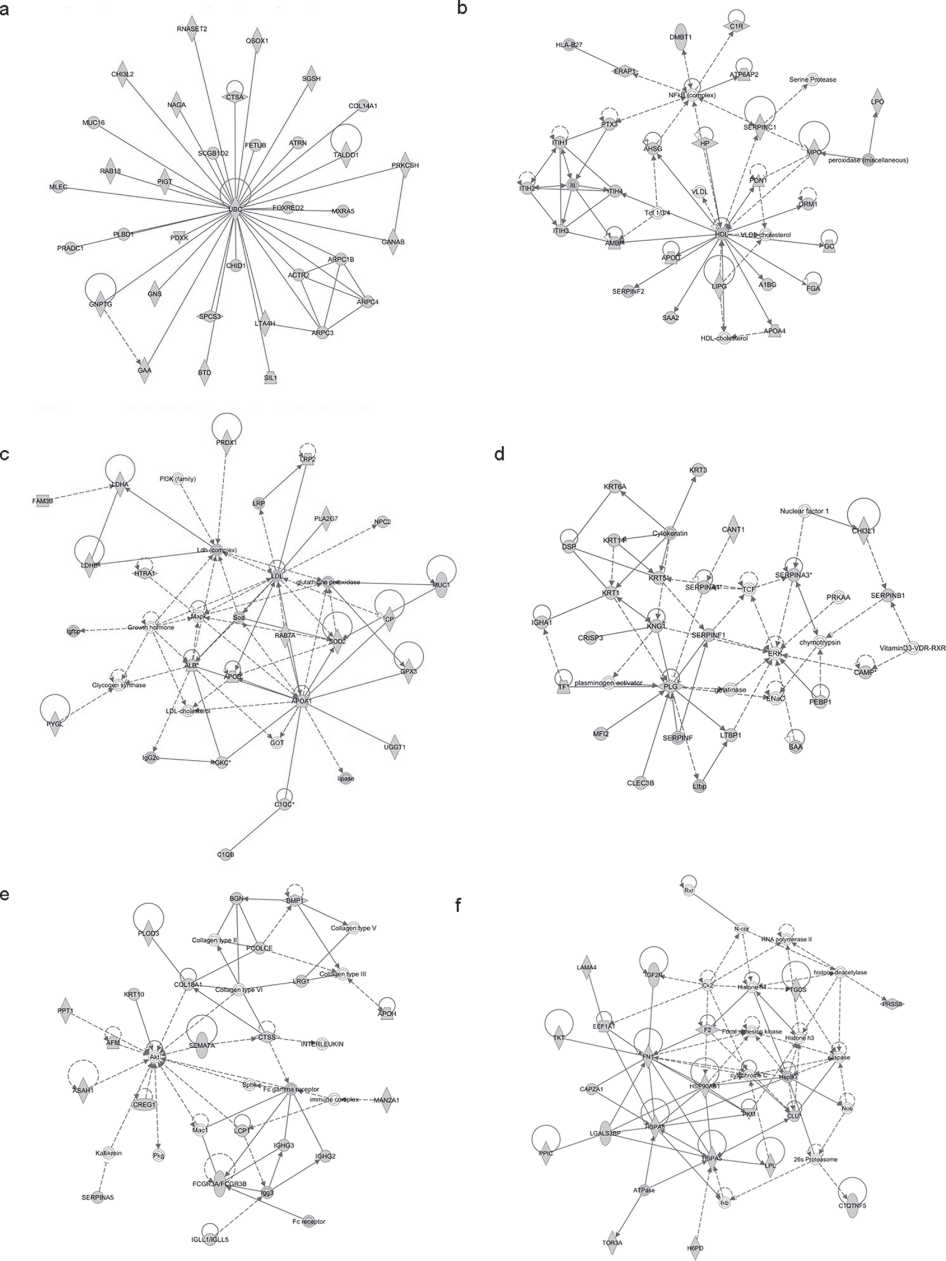
IPA analysis. In the figure are shown the top 6 signaling networks: 1) Cellular Movement, Hematological System Development and Function and Immune Cell Trafficking; 2) Cell Morphology, Cellular Movement, Digestive System Development and Function; 3) Free Radical Scavenging, Connective Tissue Disorders and Inflammatory Disease; 4) Cell-To-Cell Signaling and Interaction, Cellular Function and Maintenance, Hematological System Development and Function; 5) Organ Morphology, Skeletal and Muscular System Development and Function, Tissue Morphology; 6) Organ Morphology, Skeletal and Muscular System Development and Function, Cancer.

Identified proteins were mapped in 21 pathways of interacting protein clusters according to the identifiers' HomoloGene to the ortholog information in the Ingenuity Knowledge Base (IKB) (Fig. 3). In particular, 35 proteins involved in Developmental Disorder, Hereditary Disorder and Metabolic Disease were grouped as top 1 network, [IPA score 71 (Fig. 4A)].

The associated functions of the most interesting networks are: i) Cancer, Gastrointestinal Disease, Hepatic System Disease, [IPA score 42 (Fig. 4B)]; ii) Lipid Metabolism, Small Molecule Biochemistry, Free Radical Scavenging [IPA score 33 (Fig. 4C)]; iii); Dermatological Diseases and Conditions, Neurological Disease, Organismal Injury and Abnormalitie [IPA score 33 (Fig. 4D)]; iv) Organ Morphology, Skeletal and Muscular System Development and Function, Tissue Morphology [IPA score 33 (Fig. 4E)]; v) Organ Morphology, Skeletal and Muscular System Development and Function, Cancer [IPA score 29 (Fig. 4F)].

Obviously, the involvement of proteins in processes such as developmental disorders, metabolic diseases, and cancer, occurs when they are not correctly expressed and/or regulated.

Proteins were further categorized based on biofunction; interestingly, the most numerous groups were respectively: Cell-To-Cell Signaling and Interaction, Cellular Movement, Immune Cell Trafficking, Hematological System Development and Function, Cell Death and Survival, Free Radical Scavenging, Tissue Development, Cardiovascular System Development and Function, Organismal Development and Cellular Growth and Proliferation, as shown in S2 Supporting Information. Lastly, IPA allowed us to deduce the upstream regulator of identified proteins. Intriguingly, the molecules that regulate the major number of proteins are lipopolysaccharide, TGFB1, beta-estradiol, dexamethasone and TNF. The putative target proteins are listed in S3 Supporting Information.

Going into details on the characterization of the identified proteins in ovine whey colostrum, we found that several of them belong to the casein family (mainly alpha casein), most likely representing unassembled casein micelles. It is demonstrated that caseins are responsible for important biological functions such as ion carriers (calcium, phosphate, iron, zinc, copper), bioactive peptide precursors and immunomodulators [34]. It was also demonstrated that the casein proteolytic fragments have an antimicrobial activity, suggesting that proteases may also play a role in the host defence [35]. In addition, peptides derived from caseins are receiving much attention as possible sources of natural bioactivity with health benefits for humans, probably because they stimulate the innate immune system within the mammary gland and prevent udder infections during the dry phase [36]. As expected, proteins involved in metabolic and immune system processes account for 51.6% and 21.1% of the total proteins, respectively [36]. In our study we also found many proteins involved in lipid metabolism (fatty acid synthase, apolipoproteins A-I, butyrophilin, xanthine dehydrogenase, and endothelial lipase) suggesting that colostrum contains the enzymes necessary to balance the equilibrium between lipid synthesis and breakdown. While lipases contribute to effective digestion and retention of colostrum fat, the apolipoproteins acts as carriers of fatty acids in the circulation. Fatty acid synthase is an indispensible component of lipogenesis and energy production [37]. Xanthine dehydrogenase and butyrophilin, appears to be essential for milk fat globule production [38, 39]. Xanthine dehydrogenase and butyrophilin with other four milk fat globule membrane proteins (Milk Fat Globule-EGF factor 8 or lactadherin, ATP-binding cassette sub-family G member 2, protein disulfide isomerase family A member 3, and ras-related protein RAB11A) probably represent a small fraction dissociated from the membranes.

Particularly, butyrophilin is concentrated in the apical membranes of mammary epithelial cells and is expressed only during lactation [40]. Regarding the other four milk fat globule membrane proteins, lactadherin may play a role in the membrane vesicle secretion, such as budding or shedding of plasma membrane (micro-vesicles) and exocytosis of endocytic multivesicular bodies (exosomes) [41]. ATP-binding cassette sub-family G member 2 is included in the superfamily of ATP-binding cassette (ABC) transporters. Significant expression of this protein has been observed in the placenta, which may suggest a potential role for this molecule in this tissue. It likely serves as a cellular defense mechanism in response to xenobiotic exposure [42].

Ras-related protein RAB11A is a low molecular-weight GTP-binding proteins that coordinate stages of transport in the secretory pathway, and annexins, which include a group of calcium-dependent membrane aggregating proteins that can initiate contacts between secretory vesicle membranes, which subsequently fuse [43].

Finally, protein disulfide isomerase family A member 3 is a protein of the endoplasmic reticulum that interacts with lectin chaperones calreticulin and calnexin to modulate folding of newly synthesized glycoproteins [44].

Interestingly, we found a group of proteins involved in cellular growth and proliferation, such as transforming growth factor beta (TGF-β), and Bone morphogenetic protein 1 (BMP1).

The TGF-β belongs to a large family of proteins involved in regulating the proliferation and differentiation of many cell types, i.e. mammary gland epithelial cells or human colon adenocarcinoma Moser cells [45]. TGF-β is also a potent regulator of cell–matrix and cell-cell adhesions that plays a crucial roles in controlling the differentiation of epithelial cells and in maintaining the integrity of the epithelium [45].

BMP1 is a metallopepsidase involved in extra-cellular matrix (ECM) formation by activation a subset of the TGFβ superfamily of proteins [46].

Moreover, we identified some proteins involved in carbohydrate metabolism including gliceraldeide 3 fosfate deydrogenase, aldolase, phosphoglycerate kinase or beta 1,4-galattosyltransferase I, most of which are involved in glycolysis. We hypothesize that these proteins derive from somatic cells disrupted during whey preparation.

As stated earlier, 21.1% of proteins was identified as immune or inflammation modulators: among them, lactoferrin, bactinecin, and gelsolin isoform b were detected. Interestingly, gelsolin isoform b is described only in bovine colostrum [22] and in human milk [47]. Gelsolin, a Ca^2+^-dependent actin-regulatory protein, mapped in IPA network 4, is necessary for rapid motile responses in cell types involved in stress response and inflammation [22, 48]. Thus, the presence of gelsolin in colostrum could be related to newborns protection from noxious agents.

Other proteins of interest that play an essential role in the innate immune system are serum amyloid A (SAA) and α1-antitrypsin. SAA take part in the acute phase of inflammation: it is demonstrated that SAA concentration increases by 1000-fold within 24–48 h following infection/inflammation [36]. The role of α1-antitrypsin is to reduces the biologically active trypsin, decreasing the proteolysis. This protein has been described only in bovine colostrum where the concentration is 100 times higher than in milk. The presumed role of α1-antitrypsin is to protect immune components against the proteolytic cleavage, allowing the absorption of the intact proteins by the newborn ruminant [36].

Another group of proteins comprises proteins with anti-oxidant function, such as transferrin, lactoperoxidase, peroxiredoxin 2, superoxide dismutase (SOD2) and Glutathione peroxidase (GPx).

To the best of our knowledge, this is the most exhaustive and comprehensive report in ovine colostrum of an interesting set of proteins related with IPA network 6 and 14 (Fig. 3), and implicated in protection from stress stimuli: heat shock protein 90 (HSP90) and heat shock protein 70 (HSP70) [49–51]. These two proteins are involved in the organization of cytoskeleton and in microtubule dynamics. Moreover, Hsp70 is important in the protection and regeneration of bowel [52] and its expression is reported in sheep lung epithelial cells [53], myocardium [54], brain [55], and uterine tissue [56].

Our study, which takes advantage of a Gel-LC-MS/MS integrated approach, provides the first systematic classification of the ovine whey colostrum low abundance proteome. Data obtained from this analysis enhance our understanding of the biochemical complexity of the nutritional components of colostrum, helping to gain new insights into proteins involved in colostrogenesis and, possibly, into proteins having bioactivity in the recipient lamb with important effects on immune system and the development of gastrointestinal tract. The normal development of gastrointestinal tract will be important for the food nutrients absorption and for the growth and weight gain of the newborn.

Further studies will be necessary in order to understand how this primary source of food passes through the gastrointestinal tract of the newborn lamb how the above-mentioned regulatory proteins and peptides influence the lamb development and growth, but we are confident that this work represent a milestone in this way.

## Supporting Information

S1 Supporting Information LC-MS/MS Identification. In the table are listed identified proteins with their relative accession number, protein description, % of protein coverage, number of peptides, GO Terms, molecular weight (kDa) and isoelectric point.
(XLSX)

S2 Supporting Information IPA analysis. In the figure are shown the proteins biofunction classification. Chart were customized for molecular and cellular function and physiological system development and function.
(TIF)

S3 Supporting Information IPA analysis. In the table are listed the putative upstream regulators and their relative target.
(XLS)
