# Supplementary figures and images for: Proteomics-Driven Analysis of Ovine Whey Colostrum
Source: PLoS One. 2015 Feb 2;10(2):e0117433. doi: 10.1371/journal.pone.0117433 (PMC4313942; doi:10.1371/journal.pone.0117433)

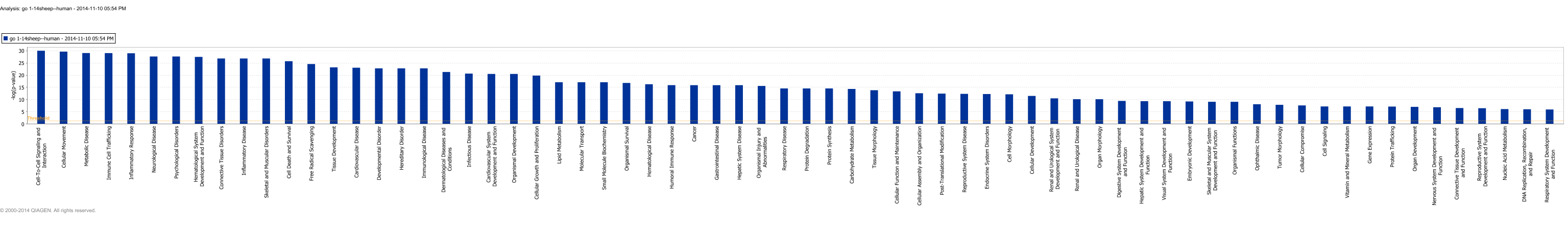

Supplement: S2 Supporting Information — In the figure are shown the proteins biofunction classification. Chart were customized for molecular and cellular function and physiological system development and function. (TIF) [file pone.0117433.s002.tif]
